# Dissecting a potential spandrel of adaptive radiation: Body depth and pectoral fin ecomorphology coevolve in Lake Malawi cichlid fishes

**DOI:** 10.1002/ece3.4651

**Published:** 2018-11-06

**Authors:** Christopher D. Hulsey, Roi Holzman, Axel Meyer

**Affiliations:** ^1^ Department of Biology University of Konstanz Konstanz Germany; ^2^ School of Zoology, Faculty of Life science Tel Aviv University, Tel Aviv, Israel and The Inter‐University Institute for Marine Sciences Eilat Israel

**Keywords:** adaptive radiation, constructional constraints, locomotion, Panglossian

## Abstract

The evolution of body shape reflects both the ecological factors structuring organismal diversity as well as an organism's underlying anatomy. For instance, body depth in fishes is thought to determine their susceptibility to predators, attractiveness to mates, as well as swimming performance. However, the internal anatomy influencing diversification of body depth has not been extensively examined, and changes in body depth could arise as a by‐product of functional changes in other anatomical structures. Using an improved phylogenetic hypothesis for a diverse set of Lake Malawi cichlid fishes, we tested the evolutionary association between body depth and the height of the pectoral girdle. To refine the functional importance of the observed substantial correlation, we also tested the coevolution of pectoral girdle height and pectoral fin area. The extensive coevolution of these traits suggests body depth in fishes like the Lake Malawi cichlids could diverge simply as a by‐product of being tightly linked to ecomorphological divergence in other functional morphological structures like the pectoral fins.

## 1 INTRODUCTION

Body depth, or the relative height of the dorsoventral body axis adjusted for body length, could be a major axis of phenotypic divergence and functional adaptation in many organisms. For instance, changes in fish body depth are commonly associated with habitat specialization (Tobler et al. 2008; Weese, Ferguson, & Robinson, 2012), trophic convergence (Krabbenhoft, Collyer, & Quattro, 2009; Ruber & Adams, 2001), and speciation (Elmer, Kusche, Lehtonen, & Meyer, 2010; Fruciano et al., 2016; Hendry & Taylor, 2004; Pfaender, Schliewen, & Herder, 2010). Changes in body depth could also influence a number of behaviors that link morphology to organismal behavior and functional abilities. For instance, the depth of the profile of the fish could determine susceptibility to predators (Abate, Eng, & Kaufman, 2010; Brönmark & Miner, 1992; Chivers, Zhao, Brown, Marchant, & Ferrari, 2008; Eklöv & Jonsson, 2007; Frommen et al., 2011; Nilsson, Brönmark, & Pettersson, 1995; Price, Friedman, & Wainwright, 2015), detection by prey (Domenici, 2002; Seamone, Blaine, & Higham, 2014; Webb, 1984a, 1984b), sexual attractiveness to mates (Head, Kozak, & Boughman, 2013), and swimming performance (Webb, 1984a, 1984b; Svanbäck & Eklöv, 2004; Domenici, Turesson, Brodersen, & Brönmark, 2008; Blob et al. 2010). However, a better understanding of what internal structures are changing when fish diverge along a body depth axis could provide improved insight into the ecological, evolutionary, and functional mechanisms structuring adaptive changes in body depth. Additionally, differences in body depth could simply arise as a “spandrel” or a phenotypic characteristic that is a by‐product of the evolution of some other characteristic, rather than a direct product of adaptive selection (Barel, 1983, 1984; Gould & Lewontin, 1979). Understanding whether particular internal anatomical traits are dictating body depth would allow us to better evaluate whether body depth might coevolve with or even arise as a by‐product of divergence in other traits. To examine one putative link between functional morphological changes underlying differences in fish body depth, we examined the evolutionary relationships between anatomical divergence in pectoral fin structure and body depth in Lake Malawi cichlid fishes.

**Figure 1:**
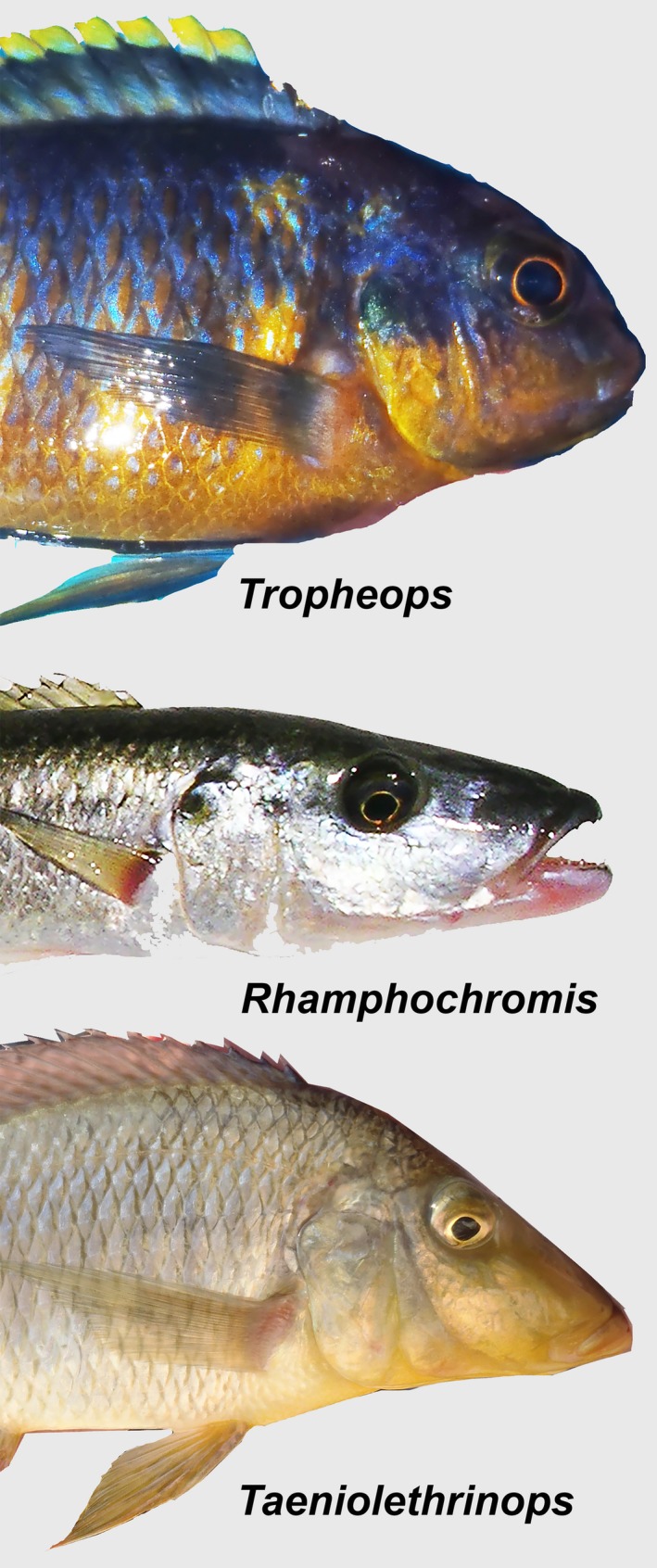
Lake Malawi cichlid representatives from three genera that display some of the diversity of body depths in this adaptive radiation

**Figure 2:**
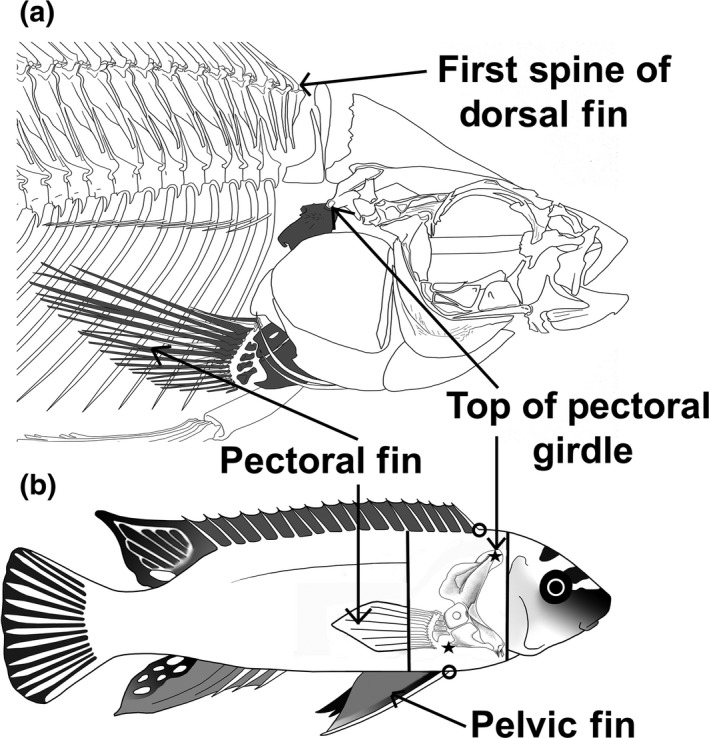
Cichlid pectoral fin morphology. In the skeleton of a generalized cichlid (a), the location of the pectoral fin and girdle that are extensively embedded within the body are highlighted in dark gray. On an image of a Malawi cichld (b), the location of several of the measurements made for this study is highlighted. For instance, we measured body depth with calipers as the distance between the first dorsal spine (dorsal circle) and the most anterior external attachment site of the pelvic fin (ventral circle). The basic morphology and position of the pectoral girdle are depicted as seen if examined internally. Subsequently, we dissected and then cleared and stained the pectoral fin and pectoral girdle. Using a digital image, we measured the height of the pectoral girdle as the distance between the dorsal‐most point on the cleithrum (dorsal star) and the posterior point on the coracoid (ventral star)

Not all phenotypic changes, even when found in adaptive radiations, are necessarily functional or adaptive. Body depth differences are a major axis of shape divergence routinely observed in morphometric studies of fish body shape (Tobler et al.^2008^; Elmer et al., 2010; Recknagel, Elmer, & Meyer, 2014; Husemann, Tobler, McCauley, Ding, & Danley, 2017) and varies considerably in Lake Malawi cichlids (Figure 1). For instance, the anterior‐most insertion of both the dorsal fin and ventrally located pectoral fin are frequently used as external morphological landmarks that closely approximate body depth (Figure 2). However, unless fish are diverging to maximize body depth itself, this measurement provides little mechanistic understanding of what advantages morphological differences in body depth confer. Body depth difference could be under strong selection, arise as the result of multivariate selection on several traits, or simply arise as a “constructional constraint,” a type of phenotypic spandrel that has diverged as a by‐product of the way that the body is constructed (Barel, 1983). Importantly, constructional constraints are thought to influence the evolution of a huge diversity of traits including aquatic insect legs (Gorb, 1995), lobster acoustic systems (Patek & Oakley, 2003), mollusk shells (Hickman, 2013; Thomas, 1988; Ubukata, Tanabe, Shigeta, Maeda, & Mapes, 2008), bryozoan colony structure (McKinney & McGhee, 2003), plant cells (Peters, Hagemann, & Tomos, 2000), and lizard skulls (Herrel, Aerts, & Vree, 2000). Constructional constraints have also commonly been invoked as mechanisms to explain divergence in the teleost and especially cichlid trophic apparatus (Arbour & López‐Fernández, 2018; Barel, 1984; de Visser & Barel, 1996; Hulsey & Hollingsworth, 2011; Hulsey, Mims, & Streelman, 2007; Smits Witte, & Povel, 1996a; Smits Witte, & Veen, 1996b). However, the possibility that teleost body depth diverges as a result of constructional constraints has not been extensively examined.

Body depth divergence must result from changes in internal structures and could diverge as a by‐product of a large number of traits. For instance, a deeper body could reflect a larger swim bladder, bigger ovaries, a bigger trophic apparatus, or more extensive lateral swimming muscles in the body wall (Blake, 2004; Camp, Roberts, & Brainerd, 2015; Camp, Scott, Brainerd, & Wilga, 2017; Campione & Evans, 2012; Carroll, Wainwright, Huskey, Collar, & Turingan, 2004; Heidhues, Swett, & Kiddy, 1961; Tytell et al., 2010; Wardle, Videler, & Altringham, 1995). However, in many teleosts groups like cichlid fishes, one obvious set of bony elements that span the dorsoventral axis of the body is the structure of the pectoral girdle (Hulsey, Roberts, Loh, Rupp, & Streelman, 2013; Thorsen & Westneat, 2005). The bones making up the pectoral girdle suspend the pectoral fin and are oriented in the same dorsoventral axis as body depth (Figure 2). The pectoral girdle often forms a rough “L”‐shaped structure with the dorsal tip located near the first dorsal spine and with the right angle of the L‐shaped pectoral girdle lying close to the ventral edge of the body (Thorsen & Westneat, 2005). Therefore, the height of the pectoral girdle that likely reflects the size of other pectoral fin structures could readily dictate body depth divergence in many fish.

The putative association between pectoral fin anatomy and body depth is important because pectoral fin phenotypic divergence has been suggested to play a role in the adaptive divergence of cichlids and other fishes. For instance, pectoral fin shape in many fishes determines swimming speeds (Bellwood & Wainwright, 2001; Fulton, Bellwood, & Wainwright, 2001; Wainwright, Bellwood, & Westneat, 2002). Pectoral fin shape is also evolutionarily correlated with a number of other external phenotypes that could be adaptive (Feilich, 2016; Larouche, Cloutier, & Zelditch, 2015). Critically, in both Malawi and Tanganyikan cichlids, larger pectoral fin muscles and fin areas are correlated and are convergently associated with feeding from the substrate as opposed to feeding in the water column (Colombo, Indermaur, Meyer, & Salzburger, 2016; Hulsey et al., 2013). Additionally, it has been experimentally shown that the number of pectoral fin beats closely tracks the number of bites Malawi cichlids take when scraping algae (Rupp & Hulsey, 2014). If body depth changes closely track the functional morphological divergence in cichlid pectoral fin morphology that has previously been shown to play a role in both trophic and habitat divergence in Malawi, this would be consistent with body depth arising merely as a constructional by‐product of adaptive pectoral fin divergence (Barel, 1983, 1984; Hulsey et al., 2007). A constructional evolutionary association between the pectoral fin morphology and body depth differences would provide a novel nonadaptive explanation for a ubiquitously measured aspect of fish morphological diversification.

To test the relationship between body depth and characteristics of Lake Malawi cichlid pectoral fins, we quantified several morphological traits within a comparative phylogenetic framework. First, we used ultra‐conserved elements to generate an improved phylogenetic hypothesis of the relationships among the 28 Malawi species examined. Then, we quantified body depth as well as pectoral girdle structure in these Lake Malawi species. Finally, we used phylogenetic independent contrasts (PICs) to determine whether changes in the musculoskeletal structure of the pectoral fins are evolutionarily associated with changes in body depth.

## 2 MATERIAL AND METHODS

### 2.1 Field collections

For morphological and phylogenetics analyses, 28 species were collected using permits from the Malawi Parks Service from a number of locations in Lake Malawi during the summer of 2010. Fish were caught using SCUBA and barrier nets. Our collections of one to five individuals per species were focused on adult males that we could diagnose based on their anatomy and coloration. Following capture, caudal fin clips were obtained and stored in 95% ETOH for subsequent DNA sequencing. Cichlid specimens were then preserved in formalin in the field and transferred to 70% ethanol in the laboratory until we could make additional dissections and measurements.

### 2.2 Phylogeny reconstruction

We combined newly generated ultra‐conserved element (UCE) sequences for eight Malawi cichlid species with data from 20 species sequenced previously (Hulsey, Zheng, Faircloth, Meyer, & Alfaro, 2017a; McGee et al., 2016). To generate the genetic data, we extracted DNA from tissues using DNEasy kits (Qiagen Inc., Germantown, MD, USA), treated extracts with RNase, and followed RNase treatment with column‐based cleanup. We then generated sequences of ultra‐conserved elements from libraries produced using a slightly modified version of the Nextera (Epicentre Biotechnologies, Madison, WI, USA) library preparation protocol for solution‐based target enrichment as detailed previously (Hulsey, Zheng, et al., 2017a). Briefly, the library preparation protocol used in vitro transposition followed by PCR to shear DNA and attach indexed sequencing adapters. Following library preparation, species‐specific libraries (500 ng) were incubated with synthetic SureSelect (Agilent Technologies, Santa Clara, CA, USA) RNA probes for 24 hr at 65°C. We followed the standard SureSelect protocol to enrich DNA libraries following hybridization and then quantified the enriched, indexed libraries using qPCR (Kapa Biosystems, Wilmington, MA, USA). Subsequently, libraries were pooled for sequencing.

We sequenced each pool of enriched DNA using single‐end 100 bp Illumina Genome Analyzer (GAIIx) runs. After sequencing, we trimmed adapter contamination, low‐quality bases, and sequences containing ambiguous base calls using a custom pipeline. Following assembly, the PHYLUCE software package (Faircloth, 2016) was implemented to align the resultant species‐specific contigs to the UCE probes used for enrichment (Faircloth, Sorenson, Santini, & Alfaro, 2013; McGee et al., 2016). After generating the relational database of matches to enriched sequences and genome‐enabled taxa, we used additional components of PHYLUCE (get_match_counts.py) to call the most common SNP for each UCE locus.

We reconstructed SNP trees using a data set that was filtered to only include the highest quality SNP per UCE locus, resulting in 1,015 SNPs. We then converted the SNP data format to FASTA via the R packages “gdsfmt” and “SNPRelate” (Zheng et al., 2012). Then, we created a Phylip interleaved alignment file using MUSCLE (Edgar, 2004) and ran the file through the PHYLIP program DNAML to infer a maximum likelihood species tree (Felsenstein, 2005). Subsequently, 1,000 non‐parametric bootstrap replicates of the maximum likelihood tree were generated using the *bootstrap.pml* function in the R package “phangorn” (Lee, Guo, & Wang, C. Kim, A. H. Paterson, 2014; Schliep, 2011). Then, 100 trees were randomly chosen to examine the phylogenetic correlations between traits.

### 2.3 Morphometrics

Upon capture in the field, the standard length (SL) of the fish was determined using dial calipers and measured to the nearest 0.1 mm. Although preservation and allometry can influence morphometric studies (Barel, 1984; Lleonart, Salat, & Torres, 2000; McCoy, Bolker, Osenberg, Miner, & Vonesh, 2006), all subsequent measurements implicitly assumed that preservation had proportionally similar effects on all individuals and that there was no substantial allometric changes across the approximately 40‐mm size range of specimens examined. We next measured in the laboratory the body depth to the nearest 0.1 mm as the length between the first dorsal spine and the anterior attachment of the pelvic fins using dial calipers. For the pectoral fin morphometrics, the right pectoral girdle of all individuals was examined. To isolate the girdle from the body, the cleithra were first separated and the right pectoral girdle freed after separating the postemporal bone from the neurocranium. The pectoral girdle was then skinned and pectoral muscles were separated from the girdle using forceps while examining the pectoral girdle under a dissecting microscope.

To measure the height of the pectoral girdle and pectoral fin area, the entire pectoral girdle connected to the fin was first placed for one day in a digestion of 5% trypsin, 30% aqueous saturated sodium borate, and 65% water. This digestion made the fins pliable. The fins were then placed for one hour in a 1% KOH aqueous solution combined with 20 mg of alcian red stain. This allowed us to readily visualize all of the pectoral fin morphology. The fins were then pinned into a naturally splayed position using water‐proof paper. A digital image of the fin with a ruler in frame for calibration was then obtained and subsequently imported into ImageJ. Using this digital image, we first measured the height of the pectoral girdle as the distance between the dorsal‐most point on the cleithrum and the posterior point on the coracoid (Figure 2). To measure fin area, a line was digitally traced from the proximal end of the dorsal‐most leading fin ray along the tips of the fin rays and then lengthwise across the radials from the proximal end of the final lagging fin ray and finally back to the proximal end of the leading fin ray. The outline enclosed the roughly circular fin, and the area thus encircled was measured as the pectoral fin area.

### 2.4 Phylogenetic comparative analyses

To perform the comparative analyses, we size standardized all measurements as their ratio to the measurements of individual SL. The linear body depth and pectoral girdle depth were readily analyzed using the linear measurements of SL. Because areas should increase as the second power of length, the square root of the fin areas was divided by each individual's SL. Using the 100 randomly chosen phylogenies, the function “pic” available in the APE package (Paradis, Claude, & Strimmer, 2004) in R was used to generate independent contrasts for the individual size‐corrected pectoral fin areas, pectoral fin height, and body depth. This function uses a Brownian motion model of trait evolution to infer character change. Finally, we used the function “cor.test” to examine the PIC correlations and their statistical significance among the three traits.

## 3 RESULTS

**Figure 3:**
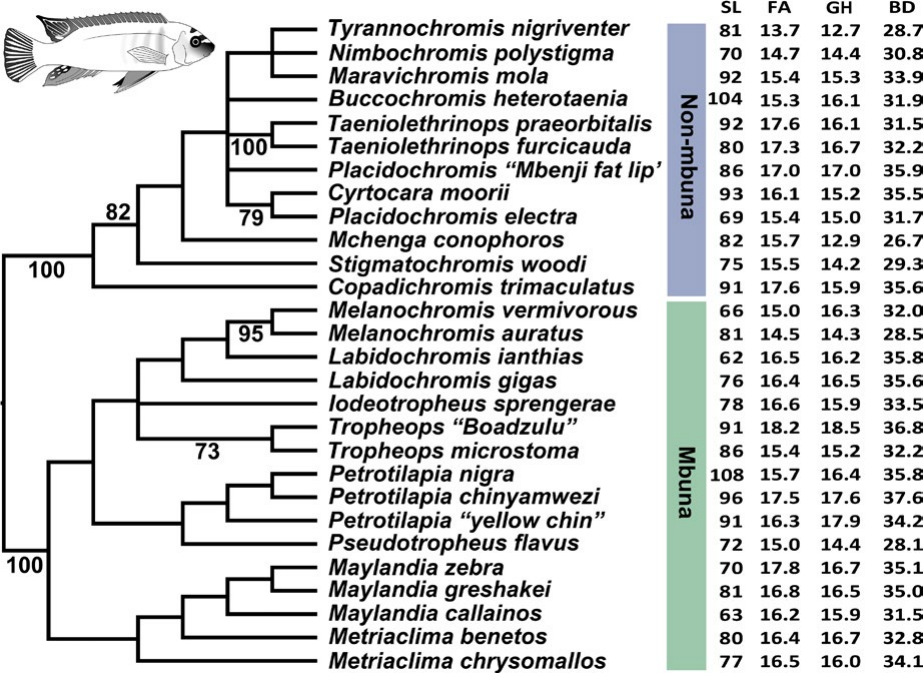
Phylogeny of 28 Malawi species reconstructed using UCE markers. The major clades of nonmbuna and mbuna are highlighted. Bootstrap values above 50% are placed behind relevant nodes. The morphometric data for each species are shown to the right of the species names. The average values of standard length (SL) for examined specimens are given first. Then, the pectoral fin area (FA) following square root transformation, pectoral girdle height (GH), and body depth (BD) is provided as a percentage of SL

As has been recovered in a number of other studies (Hulsey et al., 2007; Hulsey, Zheng, et al., 2017a; Joyce et al., 2011; McGee et al., 2016; Salzburger, Mack, Verheyen, & Meyer, 2005), the species examined fell into two major groups (Figure 3). The first group (100% bootstrap support) corresponds to a division known as the “mbuna” that includes rock‐dwelling genera like *Maylandia* and *Melanochromis*. The second major group (100%) includes nonmbuna sand‐dwelling genera like *Mchenga conophorus* and *Nimbochromis polystigma*. For the genera *Taeniolethrinops* (100%), *Melanochromis* (95%), *Tropheops* (73%), the two species sampled in each genus were recovered as monophyletic. The two *Labidochromis* species examined were closely related but recovered as paraphyletic with respect to *Melanochromis*.

**Figure 4:**
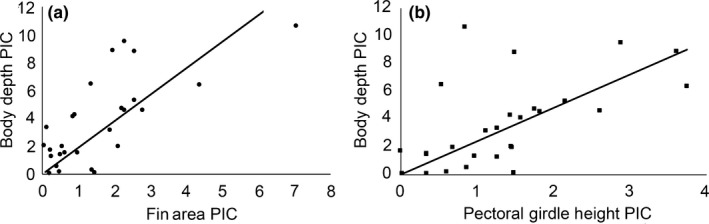
Phylogenetic independent contrast correlations of (a) pectoral fin area as well as (b) pectoral girdle height with body depth. All values were standard length size‐adjusted prior to comparative analyses on 100 randomly chosen phylogenetic reconstructions. Representative results from a single randomly chosen phylogenetic reconstruction are depicted above. Both traits were strongly correlated with body depth which is consistent with a pectoral fin‐driven constructional hypothesis for Malawi cichlid body depth divergence

Species that have evolved smaller fin areas also generally have evolved smaller pectoral girdle lengths and body depths (Figure 3). Species scattered across the Malawi cichlid phylogeny such as *Tyrannochromis nigriventer*,* Mchenga conophoros*,* Stigmatochromis woodi*, and *Melanochromis auratus* had some of the shallowest body depths, relatively small pectoral girdles, and smallest fin areas. Alternatively, disparate species such as *Cyrtocara moorii*,* Tropheops* “Boadzulu,” and *Maylandia zebra* displayed some of the greatest body depths, longest pectoral girdles, and largest pectoral fins once adjusted for SL. Following phylogenetic correction with 100 randomly chosen phylogenies to generate standard errors of estimates, the values of these three measurements were all found to be significantly correlated. Fin area evolution was highly correlated with the length of the pectoral girdle (*r* = 0.70 ± 0.04; *p* < 0.0001 ± 0.0001). Greater body depth evolution (Figure 4) was highly correlated with the evolution of both larger fin areas (*r* = 0.69 ± 0.06; *p* = 0.0003 ± 0.0010) and greater pectoral girdle height (*r* = 0.80 ± 0.03; *p* < 0.0001 ± 0.0001).

## 4 DISCUSSION

Body depth evolution in Malawi cichlids is highly correlated with divergence in pectoral morphology. Deeper bodied Malawi cichlids generally have greater pectoral girdle heights, and this greater pectoral girdle height is also associated with larger pectoral fins. Importantly, Malawi pectoral fin divergence is related to ecological divergence since fish that feed from the benthos generally have larger fin areas and muscles and use their pectoral fins intensively when feeding from the substrate (Hulsey et al., 2013; Rupp & Hulsey, 2014). Although there are undoubtedly other internal structures that contribute to body depth, the ecological opportunities and selective environments operating on the mechanical and hydrodynamic forces influencing pectoral fin diversity are likely a major determinant of body depth divergence in Malawi cichlids. Future studies of body depth divergence in this and other groups of fishes should at least consider the possibility that body depth has little adaptive value in itself and might simply reflect a constructional spandrel associated with internal anatomical structures like the pectoral girdle.

There are several potential reasons for the observed evolutionary correlation between body depth and pectoral fin ecomorphology. As seems to be the most common inference in the literature, body depth could almost always be selected for directly and provide a performance advantage for the organism that is independent of its association with pectoral fin ecomorphology. Many studies of adaptation and selection have in the past focused on single traits like body depth (Lande, 1984; Schluter, 1996). Alternatively, body depth could be the result of correlated selection on pectoral fin morphology, but still provide a performance benefit such as in swimming abilities. This type of correlational selection could potentially facilitate rapid adaptation to particular hydrodynamic regimes where a more streamlined body and smaller fins would be advantageous (Higham, 2007a; Van Wassenbergh, Potes, & Adriaens, 2018). Because correlated selection provides multiple simultaneous targets for selection, this type of integration has been suggested to play a role in the rapid evolution of structures like jaws, teeth, and a number of other traits in cichlids and other adaptively diverging groups (Albertson, Streelman, Kocher, & Yelick, 2005; Hulsey, Machado‐Schiaffino, et al., 2017b; Husemann et al., 2017). However, body depth could also be nonadaptive, provide no direct advantage to fish, and arise simply as a constructional constraint. Traits ranging from mollusk shells to the legs of waterbugs to aspects of the trophic apparatus of cichlids have all been suggested to diverge as a physical by‐product of adaptive changes in other structures (Barel, 1983, 1984 ; Gorb, 1995; Ubukata et al., 2008). It is also possible that in many cases, a fish's body depth is maladaptive such that changes in the trait come at a cost to the organism. Greater body depth could result in hydrodynamic costs for sustained swimming that arise as a by‐product of the enhanced efficacy conferred by larger fins when grazing from the substrate. The functional consequences of body depth coupled with a clearer conception of its anatomical underpinnings and their shared functional consequences for organismal performance should continue to be investigated to better understand the explicit roles of these traits during fish diversification.

Previous hypotheses of the adaptiveness of body depth might benefit from reinterpretation in light of constructional constraints, but differences in body depth could still be advantageous in groups like Malawi cichlids for several reasons (Wainwright, Alfaro, Bolnick, & Hulsey, 2005). Increased body depth could function as an impediment to gape limited predators as has been suggested for many other species (Domenici et al., 2008; Magnhagen & Heibo, 2004; Price et al., 2015). But, it would be interesting to document whether predation differs substantially between closely related species only due to body depth or whether pectoral fin morphology might simultaneously influence their predation rates. Decreased body depth could also streamline fish and result in less drag for high‐performance swimming needed to outrun predators, obtain prey, or win aggressive encounters among individuals (Fryer & Iles, 1972; Rincón, Bastir, & Grossman, 2007; Svanbäck & Eklöv, 2004; Webb, 1984a, 1984b). However, the limited cruising ranges of many Malawi cichlids suggest drag reduction during high‐speed swimming might not be very critical for many of these highly territorial and microendemic species. Locomotory influences of pectoral fins during feeding, navigating complex environments, and mating might be much more important to the fitness of these organisms (Higham, 2007a, 2007b; Hulsey et al., 2013). Regardless, body depth is likely a multifunctional trait that is in part related to pectoral fin divergence but also has its own ecological consequences (Andersson, Johansson, & Söderlund, 2006; Blake, 1983; Wainwright et al., 2005). Simultaneous testing of the effects of pectoral fin and body depth divergence across species or within phenotypically admixed individuals that can be created in hybrid crosses (Husemann et al., 2017; McGee, Reustle, Oufiero, & Wainwright, 2015) would allow a stronger parsing of the individual functional ramifications of these clearly correlated phenotypes.

Sexual selection is another area where the link between body depth and pectoral fin morphology could be important to diversification. For instance, body depth has been suggested to be a “magic” trait in sticklebacks where divergence in the presumably adaptive trait of body depth is subject to both natural and sexual selections (Head et al., 2013). Although female choice on body depth has not been examined in the Malawi radiation, female cichlids are known to show preferences for even subtle phenotypic differences in color and patterning of males (Ding et al., 2014). Therefore, it would be interesting to simultaneously test whether female Malawi cichlids show a preference for obvious phenotypic differences like deeper bodied males and/or for different sizes of pectoral fins. Preferences for ecologically relevant traits like pectoral fin size and/or body depth could provide a way to link divergent natural selection with mate preferences that should facilitate the type of ecological speciation thought to characterize Malawi cichlids (Gavrilets, 2005; Schluter & Conte, 2009; Servedio, Doorn, Kopp, Frame, & Nosil, 2011). However, these tests would be all the more effective if there was a clear link between the exact phenotypes that are mechanistically responsible for ecological divergence and the traits explicitly preferred by females.

Both body depth and pectoral fin morphology could commonly influence critical aspects of organismal diversification such as habitat specialization (Geerlink, 1983; Gerstner, 1999; Bellwood & Wainwright., 2001; Fulton et al., 2001; Higham, 2007a, 2007b; Tobler et al. 2008; Weese et al., 2012; Hulsey et al., 2013; Colombo et al., 2016), trophic convergence (Collar, Wainwright, & Alfaro, 2008; Krabbenhoft et al., 2009; Ruber & Adams, 2001; Rupp & Hulsey, 2014), and speciation (Elmer et al., 2010; Hendry & Taylor, 2004; Husemann et al., 2017; Pfaender et al., 2010). What also seems likely is that each trait individually influences a number of both independent and correlated behaviors across species that link morphology to species interactions (Brönmark & Miner, 1992; Bakker & Mundwiler, 1999; Hechter, Moodie, & Moodie, 2000; Wainwright et al., 2002; Pigliucci, 2003; Domenici et al., 2008; Blob et al. 2010; Monteiro & Nogueira, 2010; Head et al., 2013; Price et al., 2015). Further examinations of the evolutionary associations and functions of both external and internal traits will be necessary to fully understand the phenotypic bases of adaptive radiation.

## CONFLICT OF INTEREST

None declared.

## AUTHOR CONTRIBUTIONS

All authors contributed to the conception of the study. CDH collected the fishes for the study. CDH and RH collected the data morphological and genetic data as well as obtained funding for the project. All authors contributed to the writing of the manuscript.

## DATA ACCESSIBILITY

The sequences used in this study are available on Genbank (BioProject: PRJNA344532).

## References

[ece34651-bib-0001] Abate, M. E. , Eng, A. G. , & Kaufman, L. (2010). Alarm cue induces an antipredator morphological defense in juvenile Nicaragua cichlids *Hypsophrys nicaraguensis* . Current Zoology, 56, 36–42.

[ece34651-bib-0002] Albertson, R. C. , Streelman, J. T. , Kocher, T. D. , & Yelick, P. C. (2005). Integration and evolution of the cichlid mandible: The molecular basis of alternative feeding strategies. Proceedings of the National Academy of Sciences of the United States of America, 102, 16287–16292. 16251275 10.1073/pnas.0506649102 PMC1283439

[ece34651-bib-0003] Andersson, J. , Johansson, F. , & Söderlund, T. (2006). Interactions between predator‐ and diet‐induced phenotypic changes in body shape of crucian carp. Proceedings of the Royal Society B, 273, 431–437. 10.1098/rspb.2005.3343 16615209 PMC1560211

[ece34651-bib-0004] Arbour, J. , & López‐Fernández, H. (2018). Intrinsic constraints on the diversification of Neotropical cichlid adductor mandibulae size. Anatomical Record, 301, 216v226. 10.1002/ar.23713 29330955

[ece34651-bib-0005] Bakker, T. C. M. , & Mundwiler, B. (1999). Pectoral fin size in a fish species with paternal care: A condition‐dependent sexual trait revealing infection status. Freshwater Biology, 41, 543–551. 10.1046/j.1365-2427.1999.00403.x

[ece34651-bib-0006] Barel, C. D. N. (1983). Towards a constructional morphology of the cichlid fishes (Teleostei, Perciformes). Netherlands Journal of Zoology, 33, 357–424.

[ece34651-bib-0007] Barel, C. D. N. (1984). Form‐relations in the context of constructional morphology: The eye and suspensorium of lacustrine Cichlidae (Pisces, Teleostei): With a discussion on the implications for phylogenetic and allometric form‐interactions. Netherlands Journal of Zoology, 34, 439–502.

[ece34651-bib-0008] Bellwood, D. R. , & Wainwright, P. C. (2001). Locomotion in labrid fishes: Implications for habitat use and cross–shelf biogeography on the Great Barrier Reef. Coral Reefs, 20, 139–150. 10.1007/s003380100156

[ece34651-bib-0009] Blake, R. W. (1983). Median and paired fin propulsion In Webb P. W., & Weihs D. (Eds.), Fish biomechanics (pp. 214–247). New York, NY: Praeger Publishers.

[ece34651-bib-0010] Blake, R. W. (2004). Fish functional design and swimming performance. Journal of Fish Biology, 65, 1193–1222. 10.1111/j.0022-1112.2004.00568.x 20738559

[ece34651-bib-0011] Blob, R. W. , Kawano, S. M. , Moody, K. N. , Bridges, W. C. , Maie, T. , Ptacek, M. B. , ... & Schoenfuss, H. L. (2010). Morphological selection and the evaluation of potential tradeoffs between escape from predators and the climbing of waterfalls in the Hawaiian stream goby Sicyopterus stimpsoni. Integrative and Comparative Biology, 50, 1185–1199. 21558266 10.1093/icb/icq070

[ece34651-bib-0012] Brönmark, C. , & Miner, J. G. (1992). Predator‐induced phenotypical change in body morphology in crucian carp. Science, 258, 1348–1350. 10.1126/science.258.5086.1348 17778362

[ece34651-bib-0013] Camp, A. L. , Roberts, T. J. , & Brainerd, E. L. (2015). Swimming muscles power suction feeding in largemouth bass. Proceedings of the National Academy of Sciences of the United States of America, 112, 8690–8695. 10.1073/pnas.1508055112 26100863 PMC4507191

[ece34651-bib-0014] Camp, A. L. , Scott, B. , Brainerd, E. L. , & Wilga, C. D. (2017). Dual function of the pectoral girdle for feeding and locomotion in white‐spotted bamboo sharks. Proceedings of the Royal Society B: Biological Sciences, 284, 20170847 10.1098/rspb.2017.0847 PMC5543220 28724735

[ece34651-bib-0015] Campione, N. E. , & Evans, D. C. (2012). A universal scaling relationship between body mass and proximal limb bone dimensions in quadrupedal terrestrial tetrapods. BMC Evolutionary Biology, 10, 60 10.1186/1741-7007-10-60 PMC3403949 22781121

[ece34651-bib-0016] Carroll, A. M. , Wainwright, P. C. , Huskey, S. H. , Collar, D. C. , & Turingan, R. G. (2004). Morphology predicts suction feeding performance in centrarchid fishes. Journal of Experimental Biology, 207, 3873–3881. 10.1242/jeb.01227 15472018

[ece34651-bib-0017] Chivers, D. P. , Zhao, X. , Brown, G. E. , Marchant, T. A. , & Ferrari, M. C. (2008). Predator‐induced changes in morphology of a prey fish: The effects of food level and temporal frequency of predation risk. Evolutionary Ecology, 22, 561–574. 10.1007/s10682-007-9182-8

[ece34651-bib-0018] Collar, D. C. , Wainwright, P. C. , & Alfaro, M. E. (2008). Integrated diversification of locomotion and feeding in labrid fishes. Biology Letters, 4, 84–86. 10.1098/rsbl.2007.0509 18077241 PMC2412936

[ece34651-bib-0019] Colombo, M. , Indermaur, A. , Meyer, B. S. , & Salzburger, W. (2016). Habitat use and its implications to functional morphology: Niche partitioning and the evolution of locomotory morphology in Lake Tanganyikan cichlids (Perciformes: Cichlidae). Biological Journal of the Linnean Society, 118, 536–550. 10.1111/bij.12754

[ece34651-bib-0020] de Visser, J. , & Barel, C. D. N. (1996). Architectonic constraints on the hyoid's optimal starting position for suction feeding of fish. Journal of Morphology, 228, 1–18. 10.1002/(SICI)1097-4687(199604)228:1&1:AID-JMOR1&3.0.CO;2-B 29852570

[ece34651-bib-0021] Ding, B. , Daugherty, D. W. , Husemann, M. , Chen, M. , Howe, A. F. , & Danley, P. D. (2014). Quantitative genetic analyses of male color pattern and female mate choice in a pair of cichlid fishes of Lake Malawi, East Africa. PLoS ONE, 9, e114798. 25494046 10.1371/journal.pone.0114798 PMC4262453

[ece34651-bib-0022] Domenici, P. (2002). The visually mediated escape response in fish: Predicting prey responsiveness and the locomotor behaviour of predators and prey. Marine and Freshwater Behaviour and Physiology, 35, 87–110.

[ece34651-bib-0023] Domenici, P. , Turesson, H. , Brodersen, J. , & Brönmark, C. (2008). Predator‐induced morphology enhances escape locomotion in crucian carp. Proceedings of the Royal Society B: Biological Sciences, 275, 195–201. 10.1098/rspb.2007.1088 PMC2596180 17971327

[ece34651-bib-0024] Edgar, R. C. (2004). MUSCLE: Multiple sequence alignment with high accuracy and high throughput. Nucleic Acids Research, 32, 1792–1797. 10.1093/nar/gkh340 15034147 PMC390337

[ece34651-bib-0025] Eklöv, P. , & Jonsson, P. (2007). Pike predators induce morphological changes in young perch and roach. Journal of Fish Biology, 70, 155–164. 10.1111/j.1095-8649.2006.01283.x

[ece34651-bib-0026] Elmer, K. R. , Kusche, H. , Lehtonen, T. K. , & Meyer, A. (2010). Local variation and parallel evolution: Morphological and genetic diversity across a species complex of neotropical crater lake cichlid fishes. Philosophical Transactions of the Royal Society B: Biological Sciences, 365, 1763–1782 10.1098/rstb.2009.0271 PMC2871887 20439280

[ece34651-bib-0027] Faircloth, B. C. (2016). PHYLUCE is a software package for the analysis of conserved genomic loci. Bioinformatics, 32, 786–788. 10.1093/bioinformatics/btv646 26530724

[ece34651-bib-0028] Faircloth, B. C. , Sorenson, L. , Santini, F. , & Alfaro, M. E. (2013). A phylogenomic perspective on the radiation of ray‐finned fishes based upon targeted sequencing of ultraconserved elements (UCEs). PLoS ONE, 8, e65923 10.1371/journal.pone.0065923 23824177 PMC3688804

[ece34651-bib-0029] Feilich, K. L. (2016). Correlated evolution of body and fin morphology in the cichlid fishes. Evolution, 70, 2247–2267. 10.1111/evo.13021 27470271

[ece34651-bib-0030] Felsenstein, J. (2005). PHYLIP (Phylogeny Inference Package), version, 3.6. Distributed by the author Seattle, WA: Department of Genome Sciences, University of Washington.

[ece34651-bib-0031] Frommen, J. G. , Herder, F. , Engqvist, L. , Mehlis, M. , Bakker, T. C. , Schwarzer, J. , & Thünken, T. (2011). Costly plastic morphological responses to predator specific odour cues in three‐spined sticklebacks (*Gasterosteus aculeatus*). Evolutionary Ecology, 25, 641–656. 10.1007/s10682-010-9454-6

[ece34651-bib-0032] Fruciano, C. , Franchini, P. , Kovacova, V. , Elmer, K. R. , Henning, F. , & Meyer, A. (2016). Genetic linkage of distinct adaptive traits in sympatrically speciating crater lake cichlid fish. Nature Communications, 7, 12736 10.1038/ncomms12736 PMC5025864 27597183

[ece34651-bib-0033] Fryer, G. , & Iles, T. D. (1972). The Cichlid fishes of the Great Lakes of Africa. Their Biology and Evolution. Edinburgh, UK: Oliver & Boyd.

[ece34651-bib-0034] Fulton, C. J. , Bellwood, D. R. , & Wainwright, P. C. (2001). The relationship between swimming ability and habitat use in wrasses (Labridae). Marine Biology, 139, 25–33. 10.1007/s002270100565

[ece34651-bib-0035] Gavrilets , S. (2005). “Adaptive speciation” ‐ it is not that easy: a reply to Doebeli et al. Evolution, 59, 696–699.

[ece34651-bib-0036] Geerlink, P. J. (1983). Pectoral fin kinematics of *Coris Formosa* (Teleostei, Labridae). Netherlands Journal of Zoology, 33, 515–531. 10.1163/002829683X00237

[ece34651-bib-0037] Gerstner, C. L. (1999). Maneuverability of four species of coral‐reef fish that differ in body and pectoral fin morphology. Canadian Journal of Zoology, 77, 1102–1110. 10.1139/z99-086

[ece34651-bib-0038] Gorb, S. N. (1995). Design of the predatory legs of water bugs (Hemiptera: Nepidae, Naucoridae, Notonectidae, Gerridae). Journal of Morphology, 223, 289–302. 10.1002/jmor.1052230306 29865307

[ece34651-bib-0039] Gould, S. J. , & Lewontin, R. C. (1979). The spandrels of San Marco and the Panglossian paradigm: A critique of the adaptationist programme. Proceedings of the Royal Society B: Biological Sciences, 205, 581–598. 10.1098/rspb.1979.0086 42062

[ece34651-bib-0040] Head, M. L. , Kozak, G. M. , & Boughman, J. W. (2013). Female mate preferences for male body size and shape promote sexual isolation in threespine sticklebacks. Ecology and Evolution, 3, 2183–2196. 10.1002/ece3.631 23919161 PMC3728956

[ece34651-bib-0041] Hechter, R. P. , Moodie, P. F. , & Moodie, G. E. E. (2000). Pectoral fin asymmetry, dimorphism and fecundity in the Brook Stickleback, *Culaea inconstans* . Behaviour, 137, 999–1009. 10.1163/156853900502394

[ece34651-bib-0042] Heidhues, T. , Swett, W. W. , & Kiddy, C. A. (1961). Interrelationships between certain measurements of external body form, internal anatomy, and fat production. Journal of Dairy Science, 44, 115–124. 10.3168/jds.S0022-0302(61)89702-6

[ece34651-bib-0043] Hendry, A. P. , & Taylor, E. B. (2004). How much of the variation in adaptive divergence can be explained by gene flow? An evaluation using lake‐stream stickleback pairs. Evolution, 58, 2319–2331. 10.1111/j.0014-3820.2004.tb01606.x 15562693

[ece34651-bib-0044] Herrel, A. , Aerts, P. , & de Vree, F. (2000). Cranial kinesis in geckoes: Functional implications. The Journal of Experimental Biology, 203, 1415–1423. 10751157 10.1242/jeb.203.9.1415

[ece34651-bib-0045] Hickman, C. S. (2013). Interacting constraints and the problem of similarity in gastropod structure and function. American Malacological Bulletin, 31, 155–168.

[ece34651-bib-0046] Higham, T. E. (2007a). Feeding, fins, and braking maneuvers: Locomotion during prey capture in centrarchid fishes. Journal of Experimental Biology, 210, 107–117. 17170154 10.1242/jeb.02634

[ece34651-bib-0047] Higham, T. E. (2007b). The integration of locomotion and prey capture in vertebrates: Morphology, behavior, and performance. Integrative and Comparative Biology, 47, 82–95. 10.1093/icb/icm021 21672822

[ece34651-bib-0048] Hulsey, C. D. , & Hollingsworth, P. R. (2011). Do constructional constraints influence cyprinid (Cyprinidae: Leuciscinae) craniofacial coevolution? Biological Journal of the Linnean Society, 103, 136–146. 10.1111/j.1095-8312.2011.01628.x

[ece34651-bib-0049] Hulsey, C. D. , Machado‐Schiaffino, G. , Keicher, L. , Ellis‐Soto, D. , Henning, F. , & Meyer, A. 2017b The integrated genomic architecture and evolution of dental divergence in East African cichlid fishes (*Haplochromis chilotes* x *H. nyererei*). G3. 7:3195–3202. 28751505 10.1534/g3.117.300083 PMC5592944

[ece34651-bib-0050] Hulsey, C. D. , Mims, M. C. , & Streelman, J. T. (2007). Do constructional constraints influence cichlid craniofacial diversification? Proceedings of the Royal Society B: Biological Sciences, 274, 1867–1875. 10.1098/rspb.2007.0444 PMC2270932 17519189

[ece34651-bib-0051] Hulsey, C. D. , Roberts, R. J. , Loh, Y. H. E. , Rupp, M. F. , & Streelman, J. T. (2013). Lake Malawi cichlid evolution along a benthic/limnetic axis. Ecology and Evolution, 3, 2262–2272. 10.1002/ece3.633 23919168 PMC3728963

[ece34651-bib-0052] Hulsey, C. D. , Zheng, J. , Faircloth, B. C. , Meyer, A. , & Alfaro, M. E. (2017a). Phylogenomic analysis of Lake Malawi cichlid fishes: Further evidence that the three‐stage model of diversification does not fit. Molecular Phylogenetics and Evolution, 114, 44–48. 10.1016/j.ympev.2017.05.027 28579077

[ece34651-bib-0053] Husemann, M. , Tobler, M. , McCauley, C. , Ding, B. , & Danley, P. D. (2017). Body shape differences in a pair of closely related Malawi cichlids and their hybrids: Effects of genetic variation, phenotypic plasticity, and transgressive segregation. Ecology and Evolution, 7, 4336–4346. 10.1002/ece3.2823 28649345 PMC5478046

[ece34651-bib-0054] Joyce, D. A. , Lunt, D. H. , Genner, M. J. , Turner, G. F. , Bills, R. , & Seehausen, O. (2011). Repeated colonization and hybridization in Lake Malawi cichlids. Current Biology, 21, R108–R109. 10.1016/j.cub.2011.02.044 21300271

[ece34651-bib-0055] Krabbenhoft, T. J. , Collyer, M. L. , & Quattro, J. M. (2009). Differing evolutionary patterns underlie convergence in morphology in endemic fishes of Lake Waccamaw, North Carolina. Biological Journal of the Linnean Society, 98, 636–645.

[ece34651-bib-0056] Lande, R. (1984). The genetic correlation between characters maintained by selection, linkage, and inbreeding. Genetical Research, 44, 309–320. 10.1017/S0016672300026549 6530140

[ece34651-bib-0057] Larouche, O. , Cloutier, R. , & Zelditch, M. (2015). Head, body and fins: Patterns of morphological integration and modularity in fishes. Evolutionary Biology, 42, 296–311. 10.1007/s11692-015-9324-9

[ece34651-bib-0058] Lee, T.‐H. , Guo, H. , Wang, X. , Kim, C. , & Paterson, A. H. (2014). SNPhylo: A pipeline to construct a phylogenetic tree from huge SNP data. BMC Genomics, 15, 1 10.1186/1471-2164-15-162 24571581 PMC3945939

[ece34651-bib-0059] Lleonart, J. , Salat, J. , & Torres, G. J. (2000). Removing allometric effects of body size in morphological analysis. Journal of Theoretical Biology, 205, 85–93. 10.1006/jtbi.2000.2043 10860702

[ece34651-bib-0060] Magnhagen, C. , & Heibo, E. (2004). Growth in length and in body depth in young‐of‐the‐year perch with different predation risk. Journal of Fish Biology, 64, 612–624. 10.1111/j.1095-8649.2004.00325.x

[ece34651-bib-0061] McCoy, M. W. , Bolker, B. M. , Osenberg, C. W. , Miner, B. G. , & Vonesh, J. R. (2006). Size correction: Comparing morphological traits among populations and environments. Oecologia, 148, 547–554. 10.1007/s00442-006-0403-6 16604370

[ece34651-bib-0062] McGee, M. D. , Faircloth, B. C. , Borstein, S. R. , Zheng, J. , Hulsey, C. D. , Wainwright, P. C. , & Alfaro, M. E. (2016). Replicated divergence in cichlid radiations mirrors a major vertebrate innovation. Proceedings of the Royal Society B: Biological Sciences, 283, 20151413 10.1098/rspb.2015.1413 PMC4721080 26763694

[ece34651-bib-0063] McGee, M. D. , Reustle, J. W. , Oufiero, C. E. , & Wainwright, P. C. (2015). Intermediate kinematics produce inferior feeding performance in a classic case of natural hybridization. American Naturalist, 186, 807–814. 10.1086/683464 26655987

[ece34651-bib-0064] McKinney, F. K. , & McGhee, G. R. (2003). Evolution of erect helical colony form in the Bryozoa: Phylogenetic, functional, and ecological factors. Biological Journal of the Linnean Society, 80, 235–260. 10.1046/j.1095-8312.2003.00227.x

[ece34651-bib-0065] Monteiro, L. R. , & Nogueira, M. R. (2010). Adaptive radiations, ecological specialization, and the evolutionary integration of complex morphological structures. Evolution, 64, 724–744. 10.1111/j.1558-5646.2009.00857.x 19804403

[ece34651-bib-0066] Nilsson, P. A. , Brönmark, C. , & Pettersson, L. B. (1995). Benefits of a predator‐induced morphology in crucian carp. Oecologia, 104, 291–296. 28307584 10.1007/BF00328363

[ece34651-bib-0067] Paradis, E. , Claude, J. , & Strimmer, K. (2004). APE: Analyses of phylogenetics and evolution in R language. Bioinformatics, 20, 289–290. 10.1093/bioinformatics/btg412 14734327

[ece34651-bib-0068] Patek, S. N. , & Oakley, T. H. (2003). Comparative tests of evolutionary tradeoffs in a palinurid lobster acoustic system. Evolution, 57, 2082–2100. 10.1111/j.0014-3820.2003.tb00387.x 14575329

[ece34651-bib-0069] Peters, W. S. , Hagemann, W. , & Tomos, A. D. (2000). What makes plants different? Principles of extracellular matrix function in “soft” plant tissues. Comparative Biochemistry and Physiology Part A: Molecular & Integrative Physiology, 125, 151–167. 10.1016/S1095-6433(99)00177-4 10825689

[ece34651-bib-0070] Pfaender, J. , Schliewen, U. K. , & Herder, F. (2010). Phenotypic traits meet patterns of resource use in the radiation of "sharpfin" sailfin silverside fish in Lake Matano. Evolutionary Ecology, 24, 957–974. 10.1007/s10682-009-9332-2

[ece34651-bib-0071] Pigliucci, M. (2003). Phenotypic integration: Studying the ecology and evolution of complex phenotypes. Ecology Letters, 6, 265–272. 10.1046/j.1461-0248.2003.00428.x

[ece34651-bib-0072] Price, S. A. , Friedman, S. T. , & Wainwright, P. C. (2015). How predation shaped fish: The impact of fin spines on body form evolution across teleosts. Proceedings of the Royal Society B: Biological Sciences, 282, 20151428 10.1098/rspb.2015.1428 PMC4685802 26559954

[ece34651-bib-0073] Recknagel, H. , Elmer, K. R. , & Meyer, A. (2014). Crater lake habitat predicts morphological diversity in adaptive radiations of cichlid fishes. Evolution, 68, 2145–2155. 10.1111/evo.12412 24660780

[ece34651-bib-0074] Rincón, P. , Bastir, M. , & Grossman, G. S. (2007). Form and performance: Body shape and prey‐capture success in four drift‐feeding minnows. Oecology, 152, 345–355. 10.1007/s00442-006-0651-5 17277930

[ece34651-bib-0075] Ruber, L. , & Adams, D. C. (2001). Evolutionary convergence of body shape and trophic morphology in cichlids from Lake Tanganyika. Journal of Evolutionary Biology, 14, 325–332. 10.1046/j.1420-9101.2001.00269.x

[ece34651-bib-0076] Rupp, M. F. , & Hulsey, C. D. (2014). Influence of substrate orientation on feeding kinematics and performance of algae grazing Lake Malawi cichlid fishes. Journal of Experimental Biology, 217, 3057–3066. 10.1242/jeb.105080 24948641

[ece34651-bib-0077] Salzburger, W. , Mack, T. , Verheyen, E. , & Meyer, A. (2005). Out of Tanganyika: Genesis, explosive speciation, key‐innovations and phylogeography of the haplochromine cichlid fishes. BMC Evolutionary Biology, 5, 17. 15723698 10.1186/1471-2148-5-17 PMC554777

[ece34651-bib-0078] Schliep, K. P. (2011). phangorn: Phylogenetic analysis in R. Bioinformatics, 27, 592–593. 10.1093/bioinformatics/btq706 21169378 PMC3035803

[ece34651-bib-0079] Schluter, D. (1996). Adaptive radiation along genetic lines of least resistance. Evolution, 50, 1766–1774. 10.1111/j.1558-5646.1996.tb03563.x 28565589

[ece34651-bib-0080] Schluter, D. , & Conte, G. L. (2009). Genetics and ecological speciation. Proceedings of the National Academy of Sciences of the United States of America, 106, 9955–9962. 10.1073/pnas.0901264106 19528639 PMC2702799

[ece34651-bib-0081] Seamone, S. , Blaine, T. , & Higham, T. E. (2014). Sharks modulate their escape behavior in response to predator size, speed and approach orientation. Zoology, 117, 377–382. 10.1016/j.zool.2014.06.002 25041843

[ece34651-bib-0082] Servedio, M. R. , Van Doorn, G. S. , Kopp, M. , Frame, A. M. , & Nosil, P. (2011). Magic traits in speciation: ‘magic’ but not rare? Trends in Ecology & Evolution, 26, 389–397. 21592615 10.1016/j.tree.2011.04.005

[ece34651-bib-0083] Smits, J. D. , Witte, F. , & Povel, G. D. E. (1996a). Differences between inter‐ and intraspecific architectonic adaptations to pharyngeal mollusc crushing in cichlid fishes. Biological Journal of the Linnean Society, 59, 367–387.

[ece34651-bib-0084] Smits, J. D. , Witte, F. , & Van Veen, F. G. (1996b). Functional changes in the anatomy of the pharyngeal jaw apparatus of *Astatoreochromis alluaudi* (Pisces, Cichlidae), and their effects on adjacent structures. Biological Journal of the Linnean Society, 59, 389–409.

[ece34651-bib-0085] Svanbäck, R. , & Eklöv, P. (2004). Morphology in perch affects habitat specific foraging efficiency. Functional Ecology, 18, 503–518.

[ece34651-bib-0086] Thomas, R. D. K. (1988). Evolutionary convergence of bivalved shells: A comparative analysis of constructionary constraints on their morphology. American Zoologist, 28, 267–276.

[ece34651-bib-0087] Thorsen, D. H. , & Westneat, M. W. (2005). Diversity of pectoral fin structure and function in fishes with labriform propulsion. Journal of Morphology, 263, 133–150. 10.1002/jmor.10173 15549721

[ece34651-bib-0088] Tobler, M. , DeWitt, T. J. , Schlupp, I. , García de León, F. J. , Herrmann, R. , Feulner, P. G. D. , … Plath, M. (2008). Toxic hydrogen sulfide and dark caves: Phenotypic and genetic divergence across two environmental gradients in Poecilia mexicana. Evolution, 62, 2643–2649. 18637957 10.1111/j.1558-5646.2008.00466.x

[ece34651-bib-0089] Tytell, E. D. , Borazjani, I. , Sotiropoulos, F. , Baker, T. V. , Anderson, E. J. , & Lauder, G. V. (2010). Disentangling the functional roles of morphology and motion in the swimming of fish. Integrative and Comparative Biology, 50, 1140–1154. 10.1093/icb/icq057 21082068 PMC2981590

[ece34651-bib-0090] Ubukata, T. , Tanabe, K. , Shigeta, Y. , Maeda, H. , & Mapes, R. H. (2008). Piggyback whorls: A new theoretical morphologic model reveals constructional linkages among morphological characters in ammonoids. Acta Palaeontologica Polonica, 53, 113–128. 10.4202/app.2008.0108

[ece34651-bib-0091] Van Wassenbergh, S. , Potes, N. Z. , & Adriaens, D. (2018). Hydrodynamic drag constrains head enlargement for mouthbrooding in cichlids. Journal of the Royal Society, Interface, 12, 20150461. 10.1098/rsif.2015.0461 PMC4535412 26224567

[ece34651-bib-0092] Wainwright, P. C. , Alfaro, M. E. , Bolnick, D. I. , & Hulsey, C. D. (2005). Many‐to‐one mapping of form to function: A general principle in organismal design. Integrative and Comparative Biology, 45, 256–262. 10.1093/icb/45.2.256 21676769

[ece34651-bib-0093] Wainwright, P. C. , Bellwood, D. R. , & Westneat, M. W. (2002). Ecomorphology of locomotion in labrid fishes. Environmental Biology of Fishes, 65, 47–62.

[ece34651-bib-0094] Wardle, C. , Videler, J. , & Altringham, J. (1995). Tuning in to fish swimming waves: Body form, swimming mode and muscle function. Journal of Experimental Biology, 198, 1629–1636. 9319534 10.1242/jeb.198.8.1629

[ece34651-bib-0095] Webb, P. W. (1984a). Body form, locomotion, and foraging in aquatic vertebrates. American Zoologist, 28, 709–725.

[ece34651-bib-0096] Webb, P. W. (1984b). Body and fin form and strike tactics of four teleost predators attacking fathead minnow (*Pimephales promelas*) prey. Canadian Journal of Fisheries and Aquatic Sciences, 41, 157–165.

[ece34651-bib-0098] Weese, D. J. , Ferguson, M. M. , & Robinson, B. W. (2012). Contemporary and historical evolutionary processes interact to shape patterns of within‐lake phenotypic divergences in polyphenic pumpkinseed sunfish, *Lepomis gibbosus* . Ecology and Evolution, 2, 574–592. 22822436 10.1002/ece3.72 PMC3399146

[ece34651-bib-0099] Zheng, X. , Levine, D. , Shen, J. , Gogarten, S. , Laurie, C. , & Weir, B. (2012). A High‐performance computing toolset for relatedness and principal component analysis of SNP data. Bioinformatics, 28, 3326–3328. 10.1093/bioinformatics/bts606 23060615 PMC3519454

